# Engineering a hemoglobin variant with enhanced stability and oxygen affinity using a protein language model

**DOI:** 10.3389/fchem.2026.1898272

**Published:** 2026-10-05

**Authors:** Sergey Shityakov, Alexander S. Novikov, Ekaterina V. Skorb, Thomas Dandekar

**Affiliations:** 1 Laboratory of Chemoinformatics, Infochemistry Scientific Center, ITMO University, Saint Petersburg, Russia; 2 Department of Bioinformatics, Biocenter, Am Hubland, Julius-Maximilians University of Würzburg, Würzburg, Germany; 3 Department of Biology and General Genetics, Sechenov First Moscow State Medical University (Sechenov University), Moscow, Russia

**Keywords:** computational protein design, DFT calculations, hemoglobin, molecular dynamics, Monte Carlo simulation, myoglobin, oxygen affinity, protein language model

## Abstract

**Introduction:**

Engineered hemoglobin variants offer therapeutic promise for artificial blood substitutes and oxygen therapeutics, but rational design is constrained by protein stability and heme-binding. Protein language models (pLMs) predict mutation fitness, yet their integration with biophysical validation remains limited.

**Methods:**

We developed a hybrid pipeline combining pLM (ESM3) with molecular docking, Monte Carlo simulation, molecular dynamics, MM-GBSA/PBSA binding free energy calculations, and density functional theory (DFT) to engineer a stabilized hemoglobin variant (esmHb) with enhanced oxygen affinity, benchmarking against the clinically characterized Hb Fort de France mutation (α45 His→Arg).

**Results:**

ESM3 accurately assessed the fitness effect of the Hb Fort de France mutation and generated structurally stable esmHb models. Compared with the wild-type variant (wtHb), esmHb exhibited improved thermodynamic stability (ΔG: −18.48 vs. −12.22 kcal/mol), increased heme-binding affinity (ΔGbind: −11.79 vs. −8.42 kcal/mol), and greater thermodynamic favorability of oxidation according to DFT calculations (ΔE: −173.1 vs. −149.9 kcal/mol). MM-GBSA and MM-PBSA binding free energy calculations further supported improved protein–heme and protein–protein interactions, with esmHb showing more favorable values in both implicit solvation models. Statistical significance (p < 0.05) was reached for protein–protein interactions via both methods and for protein–heme interactions via MM-PBSA. We interpret the MM-GBSA results as a suggestive trend rather than definitive evidence.

**Discussion:**

This work establishes a generalizable framework for pLM-driven protein engineering applicable to therapeutic Hb variants and beyond.

## Graphical Abstract

**Figure:**
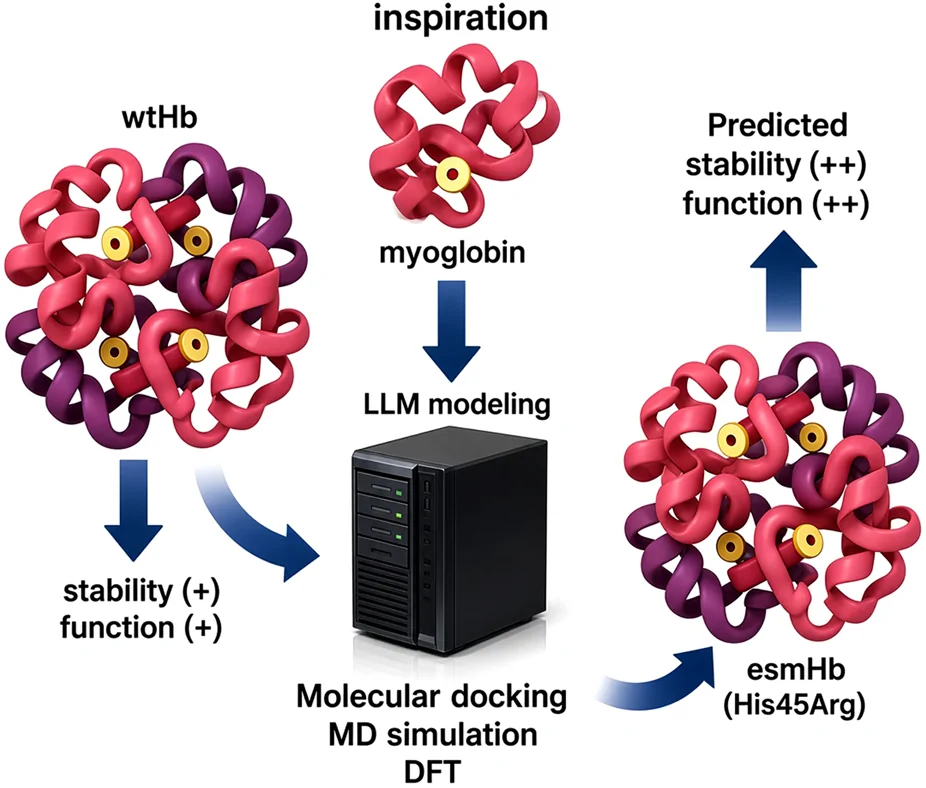

## 1 Introduction

Hemoglobin (Hb) is a critical protein in vertebrates that is responsible for oxygen transport from the lungs to tissues and facilitates carbon dioxide removal. Its tetrameric structure, which is composed of two α and two β subunits, each containing a heme group, enables efficient oxygen binding and release (Matsuhira and Sakai, 2025). However, natural Hb forms can exhibit limitations in terms of stability, oxygen affinity, or resistance to oxidative stress, prompting research into engineered variants with enhanced properties for therapeutic and biotechnological applications.

The rapid development of computational methods has opened new avenues for rationally designing proteins with customized structural and functional properties. The protein language model (pLM), which was originally developed for natural language processing, has been adapted for protein sequence and structure prediction, offering unprecedented precision in the design of novel protein variants (Gomez-Uribe et al., 2025; Li Q. et al., 2025).

The ESM generative model, in particular, has shown promise in generating protein structures by leveraging sequence and structural data to propose mutations that enhance stability and function (Fang et al., 2024; Zhang et al., 2024). In particular, the generation of a fluorescent protein (esmGFP) at a far distance (58% identity) from known fluorescent proteins mimicking over five hundred million years of evolution was promoted (Hayes et al., 2025).

However, most of the computational pipelines for protein modeling via pLM are designed not to change the sequences of protein‒ligand binding sites because of the complexity of preserving functional interactions (Lobzaev et al., 2024; Lobzaev and Stracquadanio, 2024). In addition, most of the published AI-based pipelines for protein design lack benchmarking protocols based on well-described mutations or other structural elements.

Recent advances in computational protein design, particularly through pLMs such as ESM3, offer powerful tools for *de novo* protein generation and engineering. However, the application of these models to alter ligand-binding sites remains challenging owing to the risk of disrupting functional integrity. To address this, we introduce a computational pipeline utilizing the ESM3 model for benchmarking and rational validation of a well-characterized Hb Fort de France variant [α45(CD3) His→Arg]. This approach not only computationally reproduces the enhanced structural stability and oxygen-binding affinity observed in this known high-affinity variant but also strengthens its value as a proof-of-concept for AI in validating natural mutations (Braconnier et al., 1977). By incorporating key amino acid residues inspired by the myoglobin (Mb) heme-binding site, we aimed to enhance protein‒ligand and protein‒protein interactions. Our approach combines Monte Carlo (MC) simulations, molecular dynamics, and density functional theory (DFT) calculations to evaluate the stability, binding affinities, and thermodynamic favorability of mutant (esmHb) over wild-type Hb (wtHb). This work highlights the potential of pLM-based strategies to advance rational protein design, paving the way for optimized Hb variants with properties suitable for medical and industrial applications.

## 2 Computational methods

This study employs a hybrid computational pipeline (Figure 1) that integrates rational protein design with the ESM3 generative model to validate a known high-oxygen-affinity Hb variant. The workflow begins with sequence analysis and targeted mutation (His45Arg), followed by mutant structure generation via ESM3. The subsequent steps include protein stability evaluation (FoldX, Rosetta MC), molecular docking (AutoDock), triplicate molecular dynamics (MD) simulation (AMBER v.22), and quantum mechanical analysis (DFT using ωB97XD/Gaussian-09).

**FIGURE 1:**
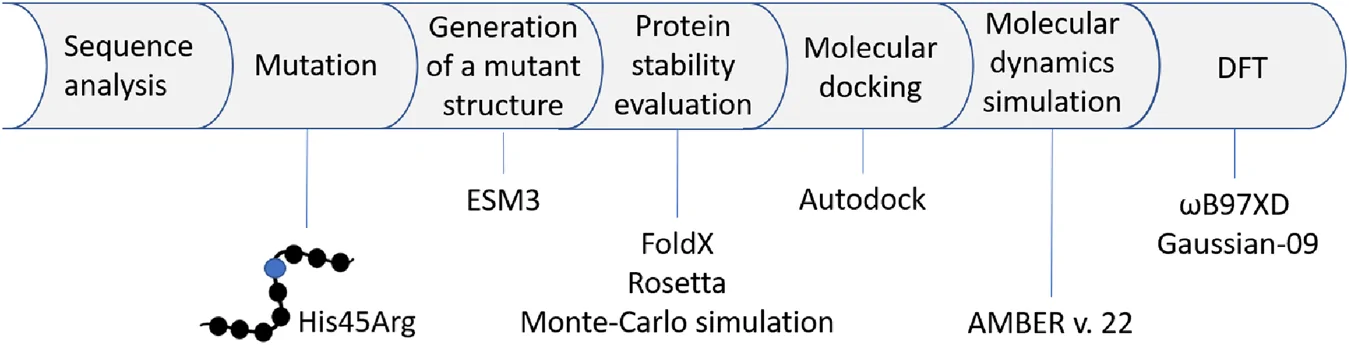
Overview of the hybrid computational pipeline for designing and validating the esmHb variant.

### 2.1 Sequence analysis and pLM-based structure prediction

Initial structural models of Hb and Mb were retrieved from the Protein Data Bank (PDB) under the identifiers 1A3N (1.8 Å resolution) and 1MBO (1.6 Å resolution), respectively (Phillips, 1980; Tame and Vallone, 2000). Sequence-level examination and manipulation were carried out via an in-house Python script. Specifically, we performed pairwise sequence alignment via pairwise aligner with the BLOSUM62 scoring matrix (gap open penalty = −10, gap extension penalty = −0.5) to map residue positions between the two proteins. The engineered residue at position 45 was modeled via the ESM3 protein language model, which was configured with a structure-focused generation strategy (temperature = 1.0). The output tokens were converted into three-dimensional backbone coordinates, which were subsequently visualized via the py3Dmol molecular viewer. The structural accuracy around the mutated region was evaluated by computing the root-mean-square deviation (RMSD), which yielded a value of 1.5 Å relative to the reference conformation. The final sequence optimization involved masking and resampling of selected positions, followed by a deterministic refolding step (temperature = 0.0) to ensure conformational consistency across the full protein.

### 2.2 Protein‒ligand docking simulations

Prior to docking, the protein structures were refined via Molecular Operating Environment version 2019.0102, and its advanced geometry optimization protocol was applied to increase the stereochemical quality and side-chain placement. Molecular docking experiments were performed via AutoDock4, which employs a semiflexible strategy in which the receptor is held rigid while the degrees of freedom of ligand torsionality are sampled. The search space was explored via the Lamarckian genetic algorithm in AutoDock4. Ligand and receptor preparation followed standard procedures: partial atomic charges were assigned via the Gasteiger method, and rotatable bonds in the ligand were automatically detected (Shityakov et al., 2021; Shityakov and Förster, 2014). The docking grid was centered on the coordinates of the cocrystallized ligand (x = −9.83 Å, y = 26.82 Å, z = 55.76 Å), with a cubic grid box of 60 × 60 × 60 Å to fully encompass the binding pocket and allow adequate ligand sampling.

### 2.3 Protein‒protein interaction analysis

FoldX software was used to predict protein‒protein affinity for wtHb and esmHb via an empirical force field algorithm for calculating free energy. It accounts for electrostatics, hydrogen bonds, van der Waals, solvation, entropy, disulfide bonds, and metal interactions, weighted by experimental data (Schymkowitz et al., 2005). Side chains were modeled with a probability-based rotamer library, and MC sampling optimizes conformations (Christensen and Kepp, 2012).

### 2.4 Monte Carlo simulation

For the evaluation of Hb protein stability, the Rosetta refinement protocol (RRID: SCR_015701) incorporating the MC approach was used. The Rosetta software is known for its ability to predict and refine protein structure. The MC algorithm predicts protein stability by matching the RMSD values with the energy scores measured in Rosetta energy units (REUs). The Boltzmann constant (kT) value of 0.8 was used as the “temperature” parameter to accept or reject moves on the basis of the Metropolis criterion (Chen and Roux, 2015). The Metropolis criterion was used to determine whether a move to a new state, in this case, a protein conformation, is accepted or rejected on the basis of the change in energy and temperature. To carry out the simulations, a “trajectory” number of 1,000 was set. The algorithm explored 1,000 different trajectories, and only the lowest-energy structures were output according to the standard protocol published elsewhere (Eirich et al., 2024).

### 2.5 Molecular dynamics simulations

Triplicate MD simulations were conducted via the AMBER v.22 software suite. Proteins were modeled with the FF99SB force field, whereas small-molecule ligands were parameterized via the generalized Amber force field (Case et al., 2005). Atomic partial charges for ligands were assigned through the AM1-BCC scheme as implemented in the antechamber utility of AmberTools, following widely accepted protocols (Marques et al., 2021). Each system was embedded in a cubic box of TIP3P explicit water molecules and charge-neutralized by introducing Na^+^ ions via the tLEap module. Electrostatic interactions beyond the cutoff distance were treated with the particle–mesh Ewald method (Essmann et al., 1995), and bonds involving hydrogen atoms were constrained via the SHAKE algorithm (Miyamoto and Kollman, 1992) to allow for a 2.0 fs integration time step. A Langevin thermostat maintained the temperature at 310 K throughout the simulations. Before production runs, the systems were energy-minimized and equilibrated over a combined 1 ns period (500,000 steps) under NVT (1.0 ns) and NPT (1.0 ns) ensembles. A final production trajectory of 500 ns was generated for each system. Binding free energies were subsequently estimated via a Python-based implementation of the MM-GBSA and MM-PBSA methodologies, which employ implicit solvation models in line with established computational protocols (Shityakov et al., 2021).

### 2.6 Quantum mechanical calculations

The ωB97XD functional was selected because it incorporates long-range corrected hybrid DFT with empirical atom–atom dispersion corrections (Chai and Head-Gordon, 2008). The Gaussian 09 software was used for the DFT calculations (Frisch et al., 2009). This combination is well suited for heme systems, where noncovalent interactions (π–π stacking between heme and proximal histidine, dispersion between the porphyrin and protein environment) and charge-transfer contributions to Fe–O_2_ bonding are both important. The range-separated nature of ωB97XD mitigates the self-interaction error that plagues local or global hybrids in transition-metal complexes with open-shell states. The CEP-121G basis sets (Stevens et al., 1984; Stevens et al., 1992) employ effective core potentials for Fe and other heavy atoms, which reduces computational cost while maintaining triple-ζ valence flexibility. To construct the DFT model systems, we extracted the heme b cofactor along with the proximal and distal coordination environment from the equilibrated MD snapshots (500 ns) of wtHb and esmHb. The model includes the iron protoporphyrin IX (heme) with the native axial ligand (His87 imidazole from the α-chain) coordinated to Fe. Residues within 5 Å of the heme were retained, but for DFT treatability, all atoms beyond the first coordination shell were truncated. Specifically, the His87 side chain was truncated at the C–C bond, retaining the imidazole ring with the N atom coordinated to Fe. The truncation was capped with a hydrogen atom placed at the C_β_ position with standard bond lengths (C–H ∼1.09 Å) and angles preserving the tetrahedral geometry of the original C_β_. The mutation site His45 → Arg45 is not directly coordinated to Fe. However, its side chain (Arg45) was retained in the truncated model down to C_β_, with the guanidinium group intact, to preserve the salt bridge with the heme 6-propionate. This allowed us to capture the electrostatic effect of the mutation on the heme–O_2_ system. All dangling bonds resulting from protein backbone truncation (e.g., at the C–C bond of residues) were saturated with hydrogen atoms (capping H atoms) via the MOE 2019.01 “Cap Termini” function with standard bond lengths and angles. No backbone atoms beyond the immediate residue side chains are present in the DFT models. The Cartesian coordinates of all the truncated models are provided in Supplementary Table S1 of the Supporting Information. The initial O_2_ geometry was derived from the MD trajectory (500 ns snapshot) for the oxy states of wtHb and esmHb. In all the cases, O_2_ was placed in the end-on (η^1^) binding mode, with the O–O bond aligned toward the distal histidine (His58 in the α-chain). The Fe–O–O angle was extracted from the MD snapshot and ranged from 118° to 124°, which is consistent with the bent geometry known for oxyhemoglobin. The O–O bond length was initially set to 1.23 Å (typical for superoxo-like Fe(III)–O_2_^–^ character) and allowed to relax during the MOE minimization protocol prior to the single-point DFT calculations. The iron spin state was explicitly considered. For deoxy models, we adopted the high-spin S = 2 (quintet) configuration (Fe(II), d^6^), which was experimentally established for deoxyhemoglobin (multiplicity = 5 for deoxy). For oxy models, we adopted the closed-shell singlet (S = 0) configuration, corresponding to the low-spin Fe(III) with a bound superoxide anion (O_2_^−^), which is the generally accepted electronic description of oxyhemoglobin (Fe(III)–O_2_^-^, S = 0, multiplicity = 1).

### 2.7 Statistical analysis

Statistical analyses were performed via GraphPad Prism 10.1.2 (GraphPad Software, Boston, MA, United States). The data are presented as the means ± SDs, as indicated. Comparisons between two groups were performed via an unpaired two-tailed Student’s t-test. Statistical significance was set at p < 0.05.

## 3 Results

### 3.1 Heme-binding site analysis

Prior to generating the ESM3 protein model, a detailed structural analysis of the heme-binding pocket in Hb revealed key active-site residues within 3 Å of the heme group (Figure 2). Among these, His87 (the proximal histidine in the α-chain) directly coordinates the heme iron and is essential for preserving both structural integrity and functional activity; thus, it cannot be substituted (Figure 2A). In contrast, His45 (the position of CE3 in the α-chain) is suitable for modification. This residue aligns with position CD3 in Mb, where arginine (Arg) naturally occupies the site and forms a robust salt bridge with the heme 6-propionate (Westrick et al., 1990), which is stronger than the polar interaction mediated by the histidine in Hb (Figure 2B). Therefore, the His45Arg mutation was implemented in the ESM3 protocol to strengthen electrostatic interactions in the heme pocket, enhancing heme retention and oxygen affinity while maintaining conserved structural features.

**FIGURE 2:**
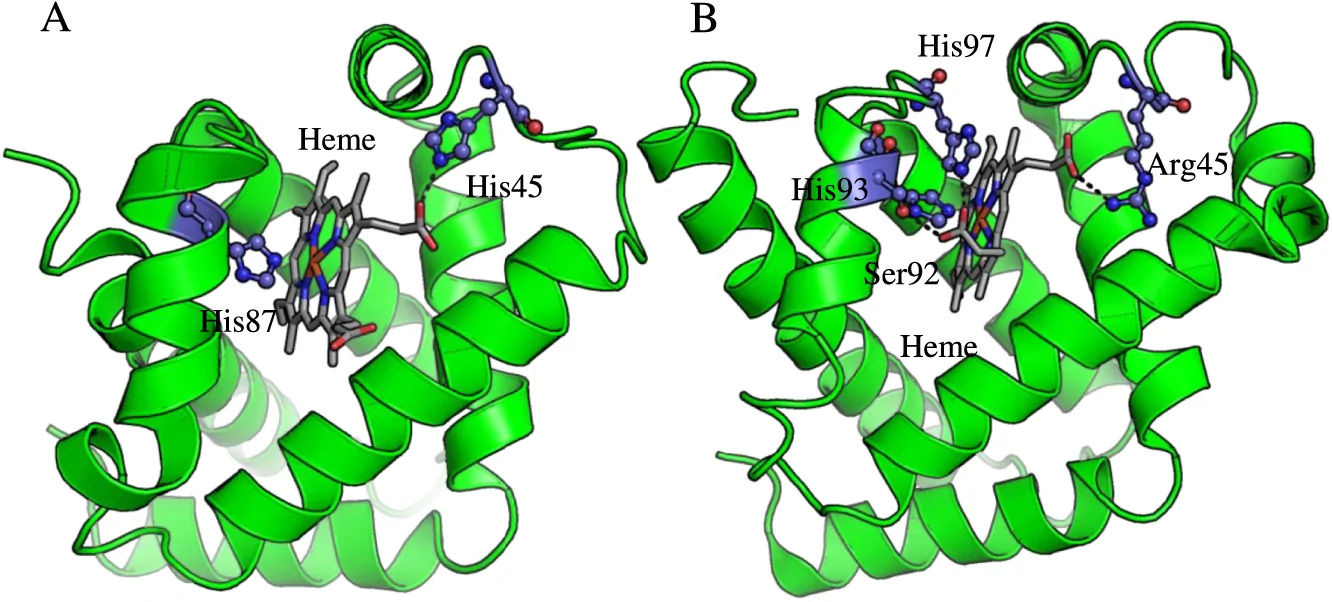
Heme-binding sites of wtHb **(A)** and Mb **(B)**. The protein is shown as a green cartoon model. The ligand (heme) is shown as a stick representation. The binding site residues are shown in ball-and-stick models. The molecules are colored according to the atom type. The H-bonds are depicted as dotted lines.

### 3.2 ESM3 mutation analysis

To evaluate the potential impact of substitutions at position 45 in the Hb α-chain, we employed the ESM3 model to predict evolutionary fitness scores for all 19 possible amino acid residues at this site. ESM3, trained on vast protein sequence and structure datasets, generates fitness estimates that reflect evolutionary conservation, functional constraints, and structural compatibility. These scores provide a quantitative measure of how likely a mutation is to maintain protein viability, with positive values indicating beneficial effects, zero values representing neutral (wild-type-like) behavior, and negative values suggesting deleterious outcomes.

As shown in Figure 3, the wild-type histidine at position 45 is assigned a fitness score of 0.0, serving as a baseline. Among all the possible substitutions, the arginine mutation presented one of the highest positive fitness scores (2.6), indicating that ESM3 predicts this change to be highly compatible with the protein’s structure and function. This high fitness is consistent with the natural occurrence of arginine at the homologous position in Mb and supports the hypothesis that this substitution is stable and functionally beneficial. Other residues, such as lysine, also received positive scores, suggesting that they are stabilizing in the core fold. Overall, the ESM3 mutation analysis supports the His45Arg variant as a functionally viable modification.

**FIGURE 3:**
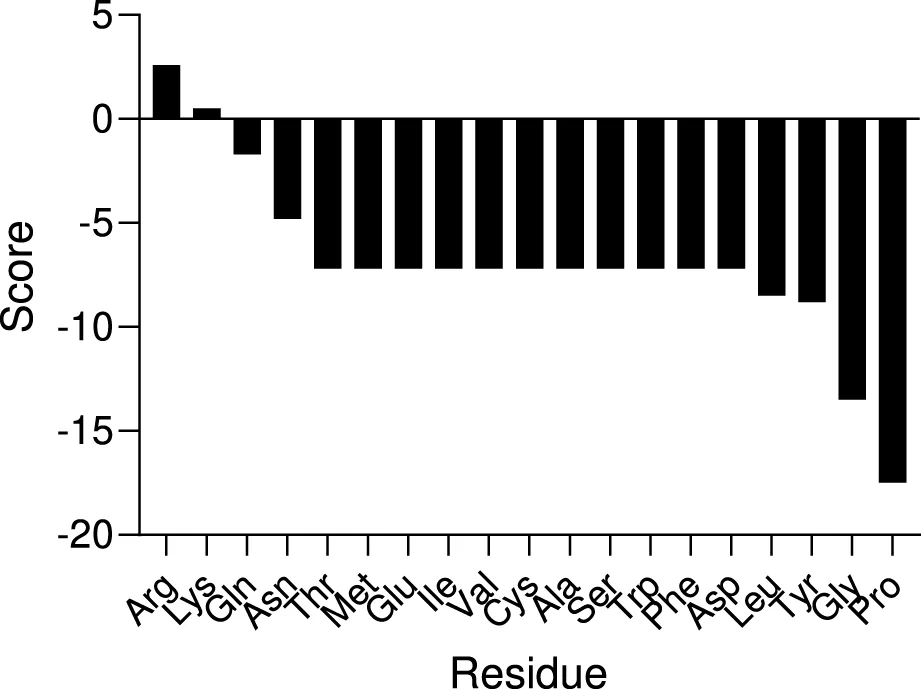
ESM3-predicted mutational effects at position 45 of the Hb α-chain. Bar plot showing fitness scores (pseudolikelihood) for 19 possible amino acid substitutions at position 45, with the wild-type histidine normalized to 0.

### 3.3 Optimization of ESM3 parameters for esmHb modeling

To optimize the ESM3 parameters for generating the esmHb variant, the human deoxy-Hb α-chain was used as the primary structural template. A systematic optimization protocol evaluated two complementary prompting strategies: (1) partial structure prompting, in which varying contiguous segments of template structure tokens (e.g., residues 1–10, 1–50, and 1–100) were retained while the remainder were masked for *de novo* generation; and (2) full template structure prompting, in which the entire template structure was provided except for local masking at the mutation site. In both strategies, position 45 (1-based numbering) was constrained to arginine to enforce the His45Arg mutation. Performance was assessed via three metrics: local RMSD at the mutation site (target < 1.5 Å), global backbone RMSD relative to the template, and postgeneration sequence identity after sequence infilling and refolding.

The analysis revealed highly nonlinear regression relationships between sequence identity and the number of retained template tokens (r^2^ = 0.97), as well as between the global backbone RMSD and the number of tokens (r^2^ = 0.96) (Figures 4A,B). The local RMSD at the mutation site (position 45) suggested improved stability with increased token retention, with a retention threshold (RT_50_) of 47.02 (Figure 4C). Here, RT_50_ is defined as the number of contiguous N-terminal structural tokens from the wild-type hemoglobin α-chain that must be retained to reduce the local RMSD at the mutation site to 50% of its baseline value observed under minimal structural prompting (i.e., near-*de novo* generation with few or no fixed tokens).

**FIGURE 4:**
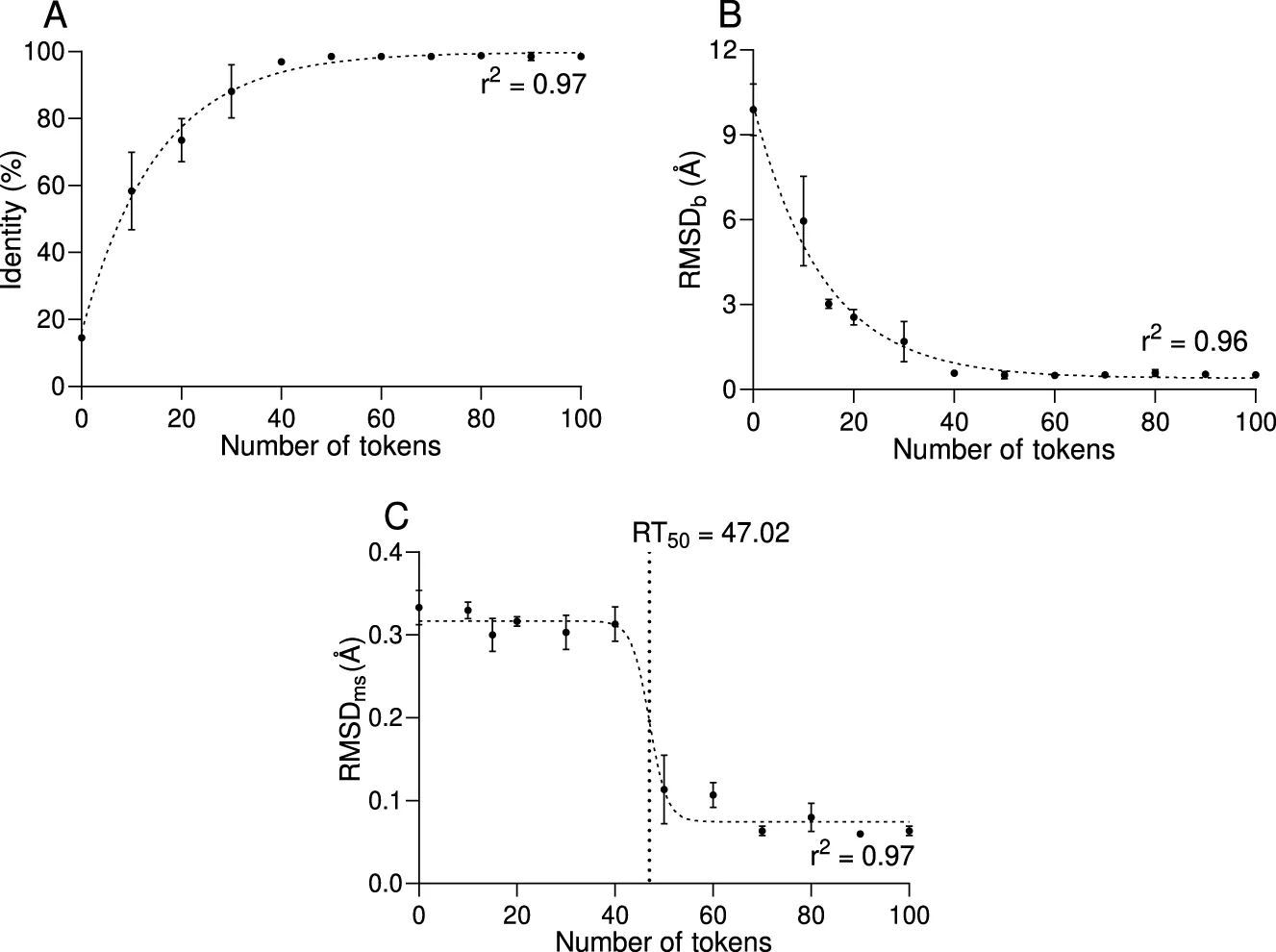
Optimization of ESM3 generation parameters for esmHb design. Sequence identity between generated and template structures as a function of retained template tokens **(A)**. Global backbone RMSD (RMSD_b_) relative to template vs. token number **(B)**. Local RMSD at the mutation site (RMSD_ms_) **(C)**. The retention threshold (RT_50_) is shown as a dotted line.

The most effective approach, retaining extensive or all nonmutated structure tokens, achieved a low local RMSD (≈1.0–1.5 Å), preserved the global fold, and ensured high sequence conservation, enabling the selection of an optimal esmHb model for downstream stability and binding analyses.

Notably, the optimized prompting strategy yielded a variant with exactly one mutation (His45Arg), achieving 100% sequence identity to the wild-type α-chain outside the targeted site. This exceptional fidelity is consistent with the ability of ESM3 for precise, single-residue engineering when guided by extensive structural templating and deterministic refolding (temperature = 0.0). This approach preserved the core globin fold while facilitating localized optimization around the mutation site.

Although the sequence identity between the human Hb α-chain and Mb is low (∼24%), structural superposition revealed strong conservation of the globin fold, particularly in the heme-binding pocket (∼40% similarity). This structural conservation provides a rational basis for introducing the Mb-derived arginine residue into Hb while preserving functional integrity.

### 3.4 Protein stability analysis

To evaluate the structural stability of the mutant, we employed FoldX software to compute the Gibbs free energy (ΔG) of protein folding for both the wtHb form and the engineered esmHb variant. The analysis indicated a ΔG of −12.22 kcal/mol for the dimeric form of wtHb, whereas the esmHb variant exhibited a more favorable ΔG of −18.48 kcal/mol. This reduction in free energy indicates the enhanced structural stability of esmHb, corroborating the effectiveness of the targeted His45Arg mutation in promoting a more stable folding conformation.

The MC simulations conducted via the Rosetta refinement protocol further supported these findings (Figures 5A–F). The algorithm explored 1,000 distinct trajectories, selecting the lowest-energy structures on the basis of the Metropolis criterion. Compared with wtHb, the esmHb variant consistently presented lower Rosetta energy scores, indicating a more energetically favorable and stable structure. These results collectively affirm that the ESM3 mutation not only preserves the essential globin fold but also improves the thermodynamic stability of the Hb variant.

**FIGURE 5:**
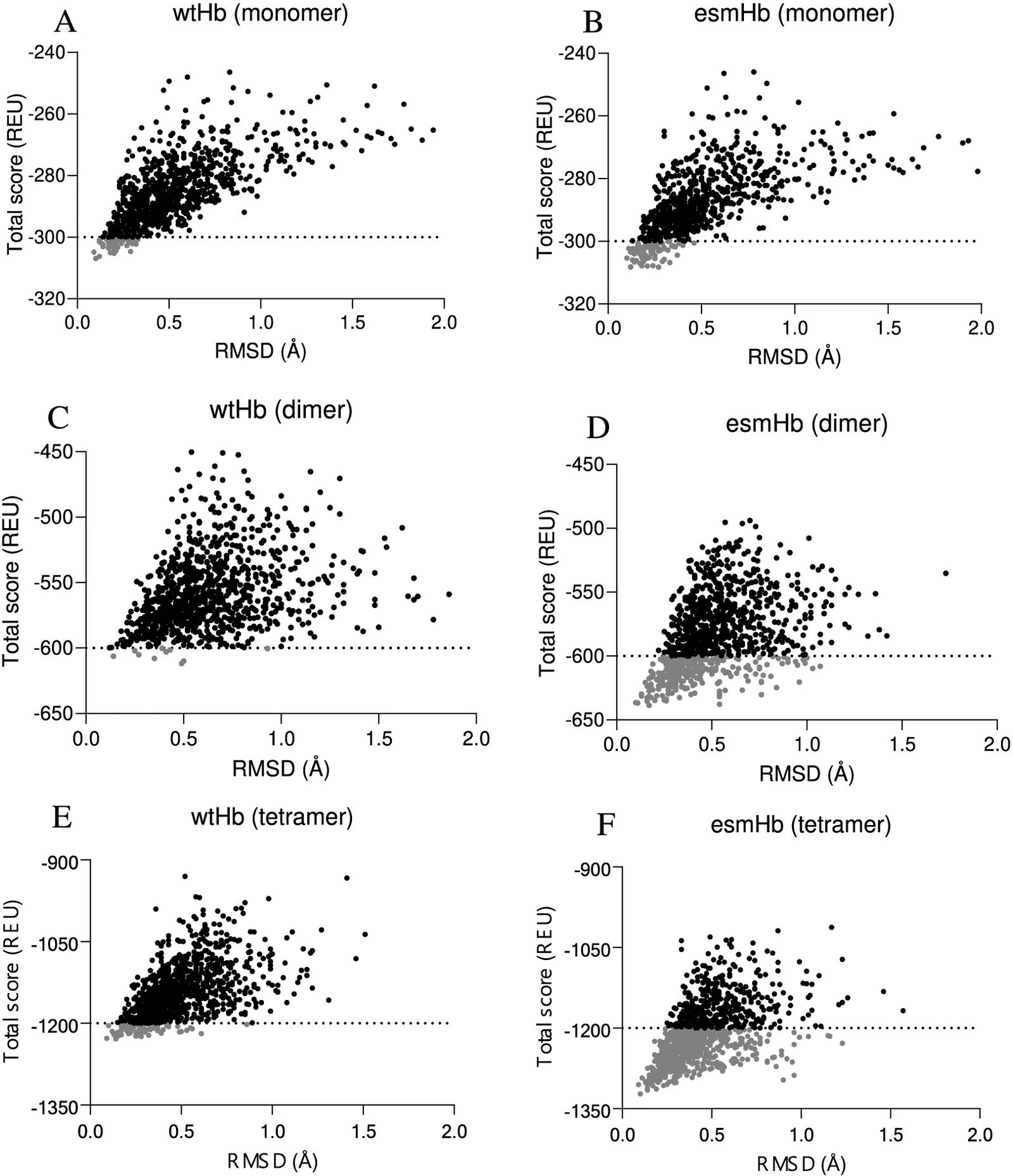
Rosetta energy landscapes for the α1 monomeric **(A,B)**, dimeric **(C,D)**, and tetrameric **(E,F)** forms of wtHb and esmHb. Each point represents a sampled conformation from 1,000 MC trajectories. The energy units are Rosetta energy units (REUs). The energy threshold is shown as a dotted line.

### 3.5 Protein‒ligand interaction analysis

The binding affinity of esmHb for heme was evaluated through molecular docking simulations via AutoDock4. The docking scores, expressed as ΔG_bind_, revealed that esmHb has a stronger interaction with heme than wtHb does. Specifically, AutoDock4 calculations provided ΔG_bind_ values of −12.4 kcal/mol for Mb, −8.42 kcal/mol for wtHb, and −11.79 kcal/mol for esmHb. These results indicate that the esmHb variant possesses a heme-binding affinity intermediate between those of Mb and wtHb but is improved relative to that of wtHb. The enhanced affinity is attributed to the introduction of arginine at position 45, which results in a more robust electrostatic interaction with the heme propionate group, analogous to the binding mechanism observed in Mb.

### 3.6 Molecular dynamics simulations

Triplicate MD simulations were performed via the AMBER v.22 software package to evaluate the dynamic stability and binding affinities of both wtHb and the ESM3-engineered variant. The heme-bound Hb dimer was used to assess protein–ligand and protein–protein interactions via various implicit solvation models, as a similar system was shown to be stable in experimental studies (Kellett, 1971).

To investigate the dynamic behavior of wtHb and the engineered esmHb variant in complex with heme, the mean RMSD, root-mean-square fluctuation (RMSF), and radius of gyration (Rg) values were analyzed and plotted versus time or residue number (Figures 6A–D). The mean protein backbone RMSD for both wtHb and esmHb relaxed after approximately 400 ns, with esmHb exhibiting marginally lower values (approximately 0.5 Å) than wtHb did, suggesting improved overall structural rigidity (Figure 6A). Block convergence analysis (Supplementary Figure S1) revealed that the wtHb trajectory reached a block error of 0.05 Å at 128 blocks, within the conventional 0.05 Å threshold, whereas the esmHb trajectory achieved full convergence at 0.04 Å. These values indicate that the ensemble averages are statistically well defined for both systems, supporting the validity of the comparative analysis. Long-range simulations (1.5–2.0 µs) would further strengthen these conclusions.

**FIGURE 6:**
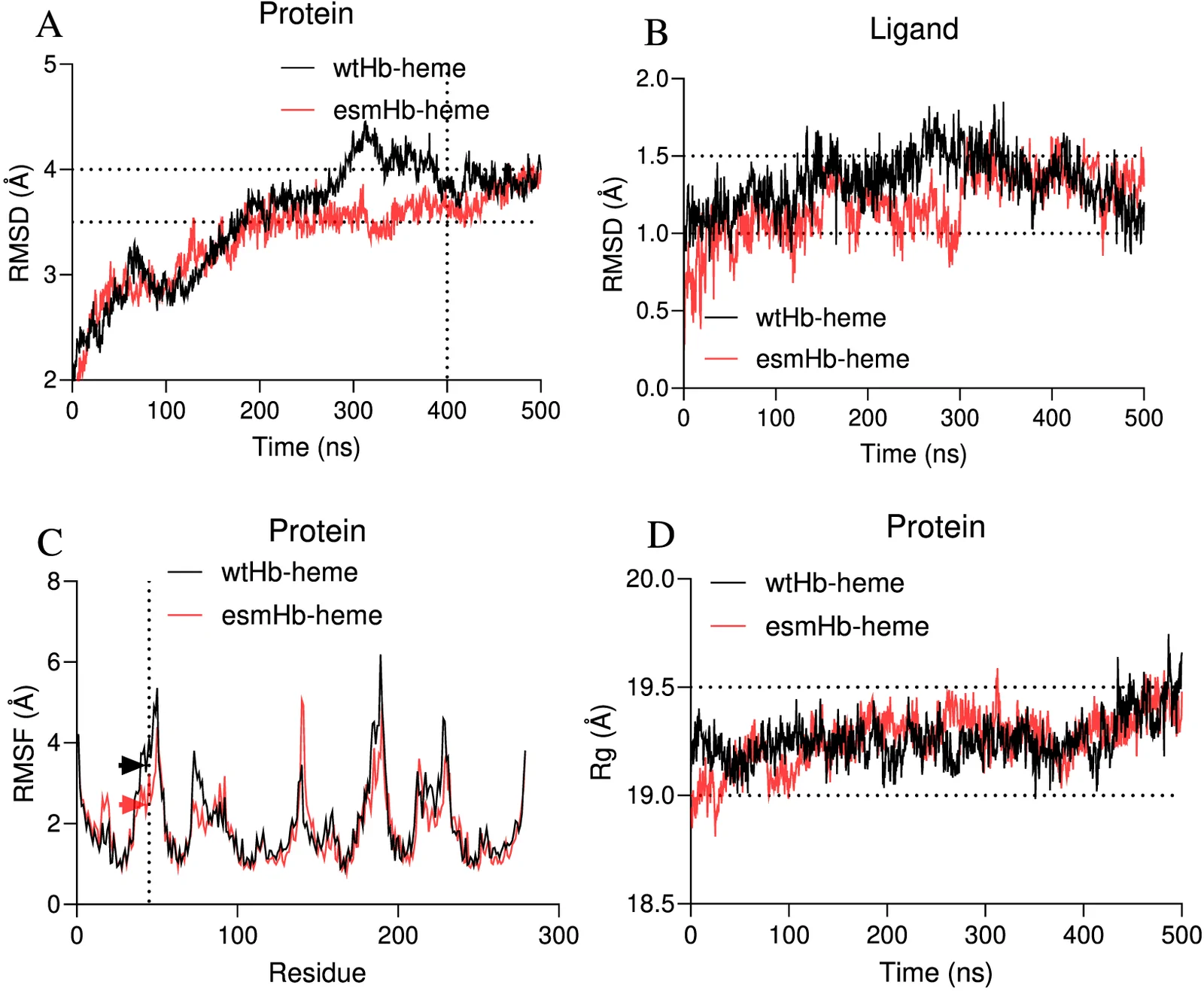
Mean RMSD **(A, B)**, RMSF **(C)**, and Rg **(D)** for the analyzed receptor (wtHb and esmHb) or ligand (heme) calculated from the 500 ns trajectories via triplicate MD. The thresholds are depicted as dotted lines. The arrows indicate the wild-type (black) and mutated (red) residues.

The RMSD of the heme ligand remained consistently low for both systems, ranging from 1.0 to 1.5 Å, with esmHb-heme showing slightly reduced deviation compared with the wtHb-heme complex, indicating improved ligand stability upon the His45Arg mutation (Figure 6B). RMSF analysis revealed that both forms displayed similar flexibility patterns across their secondary structure elements; however, esmHb showed reduced fluctuations, specifically in the heme-binding pocket region (residues surrounding position 45) where the His45Arg transition caused an *RMSF* decrease from 4.11 to 3.33 Å (Figure 6C). The Rg parameter remained stable throughout the simulation for both complexes, with esmHb maintaining a slightly more compact conformation (∼19.0 Å) than wtHb (∼19.5 Å), indicating enhanced global compactness and structural integrity in the engineered variant (Figure 6D).

Binding free energies were calculated from the production runs via the MM-GBSA and MM-PBSA methods using the trajectories from the triplicate MD simulations. With respect to protein–heme interactions, esmHb showed improved binding over wtHb (MM-GBSA: −30.22 ± 4.04 vs. −22.98 ± 2.05 kcal/mol; MM-PBSA: −64.21 ± 4.25 vs. −53.95 ± 3.03 kcal/mol), with only MM-PBSA reaching statistical significance (p = 0.03), likely attributable to the Arg45–heme propionate salt bridge (Figure 7A). We interpret this as suggestive rather than definitive evidence. Protein–protein binding affinities were also greater for esmHb (MM-GBSA: −62.94 ± 2.78 vs. −56.15 ± 0.78 kcal/mol; MM-PBSA: −146.78 ± 7.74 vs. −132.20 ± 3.03 kcal/mol), with both methods being statistically significant (p = 0.04; Figure 7B).

**FIGURE 7:**
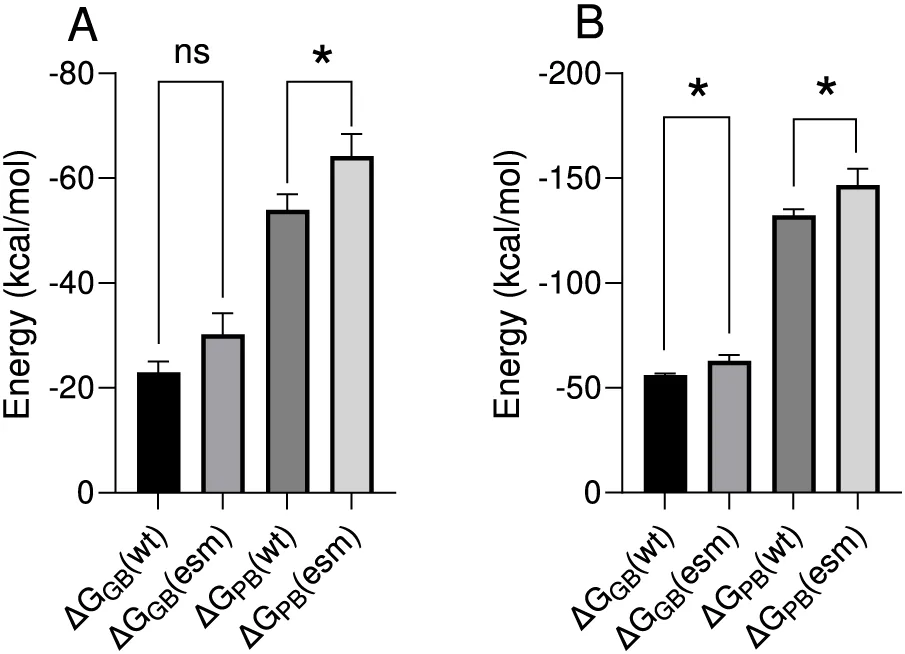
Comparative MM-GBSA (ΔG_GB_) and MM-PBSA (ΔG_PB_) binding free energy profiles for protein–heme **(A)** and protein–protein **(B)** interactions in wtHb and esmHb complexes. The data represent the means ± SDs from three independent experiments (n = 3). Statistical significance was assessed via unpaired two-tailed Student's t-tests. Statistically significant differences between wtHb and esmHb are indicated (*p < 0.05); ns = not significant.

### 3.7 Free energy landscape analysis

The free energy landscapes (FELs) derived from principal component analysis (PCA) of 500 ns molecular dynamics trajectories revealed distinct conformational dynamics between wtHb (Figures 8A,B) and the engineered esmHb variant (Figures 8C,D). The FEL of esmHb displays a single, well-defined energy basin, indicating that the protein predominantly samples a narrow and stable conformational ensemble. In contrast, wtHb has a broader and more complex energy landscape characterized by a wider distribution of low-energy states and the presence of multiple shallow minima. This finding suggests that the wild-type structure introduces additional conformational flexibility, allowing the protein to sample a larger conformational space than the mutant.

**FIGURE 8:**
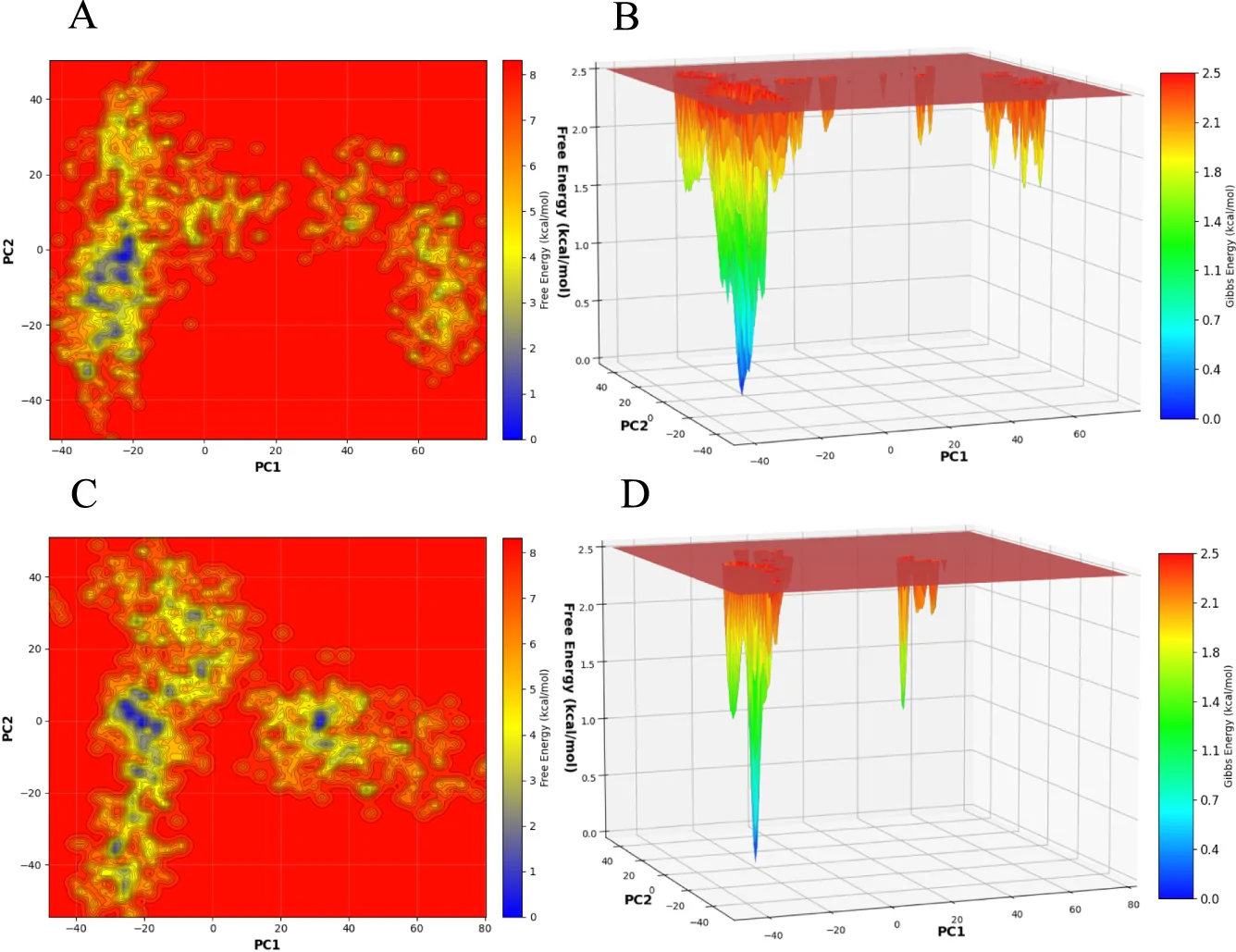
2D and 3D FELs of wtHb **(A, B)** and esmHb **(C, D)** dimers bound to heme.

### 3.8 DFT analysis

The DFT analysis of such large, real biochemical systems is extremely time-consuming and computationally intensive. Therefore, to evaluate the thermodynamic favorability of wtHb vs. esmHb oxidation, only the binding site amino acids representing the protein were used as a standard DFT protocol (Lindahl and Friedman, 2024; Schulze and Cafiero, 2024).

In particular, we carried out single-point DFT calculations at the ωB97XD/CEP-121G level of theory on the basis of the results of the preliminary geometry optimization procedure via the MOE minimization protocol for simplified model systems (see the computational details section). The results of the DFT calculations revealed that the formal oxidation reaction in the case of esmHb is thermodynamically more profitable than that in the case of wtHb by 23.2 kcal/mol (Tables 1, 2; Figure 9).

**TABLE 1:** Calculated values of total electronic energies for model structures (single-point DFT calculations at the ωB97XD/CEP-121G level of theory on the basis of the results of the preliminary geometry optimization procedure via the MOE minimization protocol).

Model structure	E, a.u
Deoxy-esmHb	−595.597242812
Oxy-esmHb	−627.655725016
O_2_ (esmHb)	−31.7826138366
Deoxy-wtHb	−595.605740699
Oxy-wtHb	−627.627264308
O_2_ (wtHb)	−31.7826036655

**TABLE 2:** Calculated values of ΔE for formal oxidation reactions.

Formal oxidation reaction	ΔE, kcal/mol
Deoxy-esmHb + O_2_ → oxy-esmHb	−173.1
Deoxy-wtHb + O_2_ → oxy-wtHb	−149.9

**FIGURE 9:**
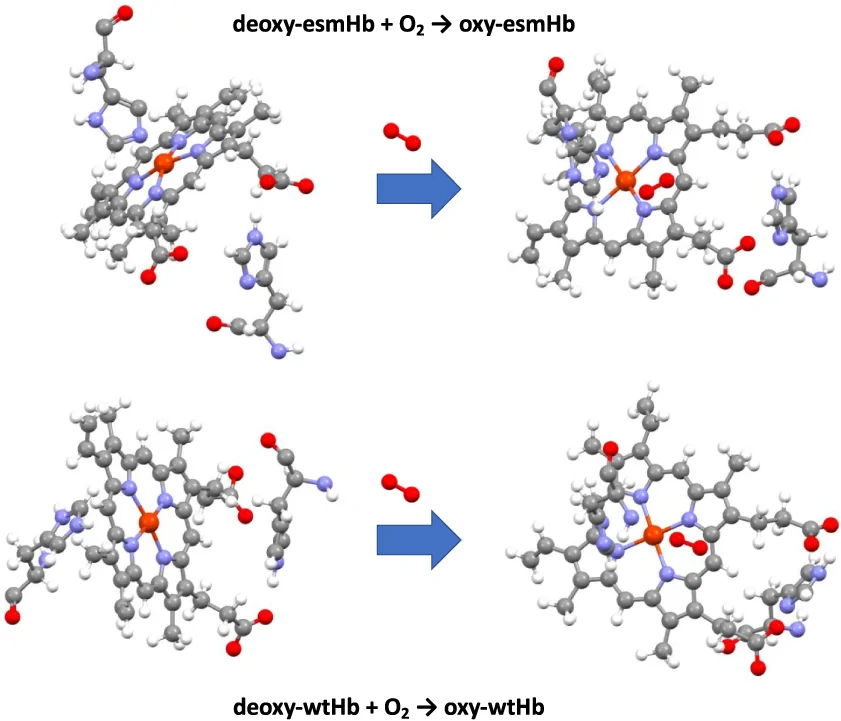
Simplified model systems for DFT calculations. Dangling bonds from the protein backbone and side chain truncations are capped with hydrogen atoms (shown as small white spheres). The heme iron (orange sphere) is coordinated to the imidazole ring of His87 (truncated at Cβ) and, in oxy models, to O_2_ (red spheres) in the bent end-on geometry. The His45 → Arg45 mutation (esmHb) includes the guanidinium group of Arg45.

## 4 Discussion

This study demonstrates the potential of integrating ESM3 with biophysical simulations to engineer esmHb with enhanced stability and function. By introducing the His45Arg substitution, inspired by the homologous position in Mb, we improved the structural stability, heme-binding affinity, and thermodynamic favorability of oxidation.

### 4.1 Advances in pLM-driven protein design

Protein language models such as ESM3 enable precise protein engineering by processing and generating sequences, structures, and functions (Tule et al., 2024; Yang et al., 2025). Integrating pLMs with traditional computational methods, such as MD simulations and DFT, has expanded the scope of protein engineering, allowing the evaluation of dynamic stability, binding affinities, and thermodynamic properties (Agoni et al., 2025; Buehler, 2024; Imam et al., 2025). Our study corroborates this synergy, as esmHb exhibited improved heme retention and oxygen affinity according to both MD and DFT analyses. It is important to note that the AutoDock4 docking scores (ΔG_bind_ of −8.42 kcal/mol for wtHb and −11.79 kcal/mol for esmHb) and the MM-GBSA/MM-PBSA binding free energies derived from triplicate MD trajectories provide complementary information. While both approaches consistently indicate enhanced heme affinity for esmHb, they should be interpreted as supportive evidence rather than as directly comparable quantitative measures.

### 4.2 Structural stability and functional enhancement

The enhanced stability of esmHb, as evidenced by a lower ΔG, underscores the efficacy of the His45Arg mutation, which likely introduces a robust salt bridge with the heme 6-propionate, as seen in Mb (Westrick et al., 1990). Rosetta MC simulations further validated this stability, demonstrating the potential of esmHb for artificial blood substitutes and oxygen therapeutics (Gomez-Uribe et al., 2025). The free energy landscapes revealed a more compact conformational ensemble for esmHb, indicating reduced conformational entropy and enhanced kinetic stability, which is consistent with recent pLM-based stability analyses (Li X. et al., 2025). Our computational findings align with experimental data on the naturally occurring Hb Fort de France variant (α45 His→Arg). Braconnier et al. (1977) first described its increased oxygen affinity, and subsequent NMR studies confirmed its structural integrity despite functional enhancement (Perutz et al., 1985). This phenotype corroborates our DFT results, which indicate 23.2 kcal/mol greater thermodynamic favorability for esmHb oxidation (ΔE: −173.1 vs. −149.9 kcal/mol). The mechanistic basis is illuminated by Mb studies: Arg45 (CD3) modulates geminate recombination and oxygen affinity in proportion to its basicity (Carver et al., 1991) and participates in a dynamic “molecular doorstop” mechanism facilitating ligand access and retention (Ringe et al., 1984). These parallels validate the ability of ESM3 to rediscover and rationalize beneficial mutations, bridging computational predictions with empirical observations.

### 4.3 Implications for therapeutic and industrial applications

The successful design of esmHb opens avenues for optimized Hb variants with tailored properties. Integrating ESM3 with experimental benchmarking could accelerate the discovery of protein-based therapeutics for hemoglobinopathies and oxygen delivery systems. Beyond Hb engineering, ESM3-driven design holds promise for enzyme engineering, drug design and discovery, and sustainable biomaterials (Yang et al., 2025). As the field evolves, combining AI-driven design with experimental benchmarking will be critical for translating computational predictions into real-world solutions.

### 4.4 Limitations of ESM3 and future directions

ESM3, trained on vast datasets, may inherit biases that overemphasize well-represented protein families while underperforming on novel motifs (Ding and Steinhardt, 2024). Additionally, its reliance on statistical patterns rather than explicit physical simulations may overlook subtle quantum effects or long-range interactions. This explains why most AI pipelines, including ours, cautiously approach ligand-binding site modifications. In our case, the His45Arg mutation was inspired by Mb homology rather than purely *de novo* generation, highlighting a limitation in the ability of ESM3 to innovate high-risk changes without structural disturbance. Experimental benchmarking of oxygen-binding kinetics and *in vivo* stability is essential to confirm therapeutic potential, as computational metrics do not fully replicate physiological complexities such as allosteric effectors (Lal et al., 2017). Future optimizations could involve fine-tuning ESM3 parameters for specific protein classes or integrating hybrid physics-based models.

## 5 Conclusion

This study demonstrates that integrating the ESM3-based pLM with biophysical simulations provides a robust framework for engineering hemoglobin variants with enhanced stability and function. Compared with wtHb, the engineered esmHb variant, featuring the His45Arg mutation, tended toward improved thermodynamic stability, heme-binding affinity, and oxidation favorability, as validated through MD simulations, binding free energy calculations, and DFT analysis. Our pipeline successfully navigated the challenge of modifying a ligand-binding site, an area typically avoided in AI-driven design, by combining evolutionary fitness prediction with structural templating and multiscale computational validation. While experimental validation remains essential to confirm therapeutic potential, this work establishes a generalizable approach for pLM-driven protein engineering that is applicable to hemoglobinopathies, artificial blood substitutes, and more.

## Data Availability

The datasets presented in this study can be found in online repositories. The names of the repository/repositories and accession number(s) can be found below: https://github.com/virtualscreenlab/ESM3-Hb.
