# Supplementary material for: Engineering a hemoglobin variant with enhanced stability and oxygen affinity using a protein language model
Source: Front Chem. 2026 Oct 5;14:1898272. doi: 10.3389/fchem.2026.1898272 (PMC13678298; doi:10.3389/fchem.2026.1898272)
Supplement: Supplementary file 1 [file Supplementaryfile1.docx]

**Supporting Information**

**Table S1**. Cartesian atomic coordinates for model structures used for DFT calculations.

| Atom | X | Y | Z |
| --- | --- | --- | --- |
| **deoxy-esmHb** | | | |
| N | 40.618000 | 28.193000 | 26.549000 |
| C | 41.289000 | 28.318000 | 25.247000 |
| C | 40.669000 | 29.433000 | 24.388000 |
| O | 41.382000 | 30.293000 | 23.866000 |
| C | 41.292000 | 26.945000 | 24.549000 |
| C | 42.441000 | 26.731000 | 23.591000 |
| C | 42.418000 | 25.912000 | 22.501000 |
| N | 43.742000 | 27.214000 | 23.763000 |
| C | 44.466000 | 26.683000 | 22.759000 |
| N | 43.698000 | 25.897000 | 21.994000 |
| H | 39.958000 | 27.431000 | 26.686000 |
| H | 42.326000 | 28.601000 | 25.428000 |
| H | 40.347000 | 26.796000 | 24.025000 |
| H | 41.375000 | 26.163000 | 25.305000 |
| H | 41.577000 | 25.334000 | 22.144000 |
| H | 45.527000 | 26.839000 | 22.598000 |
| H | 44.043000 | 25.301000 | 21.232000 |
| N | 46.038000 | 28.965000 | 11.124000 |
| C | 44.903000 | 28.283000 | 10.501000 |
| C | 45.281000 | 27.568000 | 9.184000 |
| O | 45.040000 | 26.368000 | 9.028000 |
| C | 43.718000 | 29.244000 | 10.325000 |
| C | 42.923000 | 29.419000 | 11.598000 |
| C | 42.099000 | 28.687000 | 12.530000 |
| N | 43.255000 | 30.588000 | 12.308000 |
| C | 42.737000 | 30.350000 | 13.702000 |
| N | 41.968000 | 29.268000 | 13.715000 |
| H | 45.916000 | 29.936000 | 11.394000 |
| H | 44.563000 | 27.502000 | 11.178000 |
| H | 43.042000 | 28.837000 | 9.585000 |
| H | 44.041000 | 30.195000 | 9.923000 |
| H | 44.180000 | 31.036000 | 12.197000 |
| H | 42.835000 | 31.002000 | 14.527000 |
| C | 44.112000 | 29.482000 | 19.689000 |
| C | 37.631000 | 33.520000 | 13.552000 |
| C | 37.073000 | 25.757000 | 12.570000 |
| C | 43.017000 | 24.586000 | 17.624000 |
| N | 41.917000 | 29.837000 | 16.698000 |
| C | 45.604000 | 29.819000 | 19.649000 |
| C | 36.882000 | 34.495000 | 14.104000 |
| C | 35.742000 | 25.633000 | 12.398000 |
| C | 42.296000 | 24.318000 | 18.955000 |
| N | 39.948000 | 31.035000 | 14.955000 |
| C | 46.090000 | 29.927000 | 21.069000 |
| C | 43.134000 | 23.597000 | 19.980000 |
| N | 41.180000 | 27.211000 | 15.720000 |
| C | 42.851000 | 27.689000 | 17.463000 |
| C | 41.373000 | 32.239000 | 16.571000 |
| C | 38.193000 | 30.538000 | 13.302000 |
| C | 39.571000 | 25.987000 | 14.307000 |
| C | 43.477000 | 32.431000 | 18.900000 |
| C | 39.744000 | 34.701000 | 15.614000 |
| C | 36.295000 | 28.679000 | 11.686000 |
| C | 40.863000 | 23.498000 | 15.574000 |
| C | 42.707000 | 29.066000 | 17.537000 |
| C | 40.369000 | 32.182000 | 15.610000 |
| C | 38.252000 | 29.149000 | 13.312000 |
| C | 40.610000 | 26.052000 | 15.218000 |
| O | 46.420000 | 28.896000 | 21.686000 |
| O | 42.590000 | 22.733000 | 20.698000 |
| C | 43.320000 | 29.922000 | 18.477000 |
| C | 39.587000 | 33.264000 | 15.154000 |
| C | 37.393000 | 28.266000 | 12.631000 |
| C | 41.236000 | 24.946000 | 15.829000 |
| O | 45.912000 | 31.003000 | 21.671000 |
| O | 44.337000 | 23.918000 | 20.111000 |
| C | 42.981000 | 31.194000 | 18.178000 |
| C | 38.684000 | 32.791000 | 14.252000 |
| C | 37.734000 | 26.994000 | 12.957000 |
| C | 42.158000 | 25.415000 | 16.695000 |
| C | 42.069000 | 31.161000 | 17.098000 |
| C | 38.916000 | 31.399000 | 14.109000 |
| C | 38.870000 | 27.065000 | 13.795000 |
| C | 42.120000 | 26.828000 | 16.658000 |
| N | 39.165000 | 28.400000 | 14.033000 |
| Fe | 40.719000 | 29.141000 | 15.168000 |
| H | 43.655000 | 29.975000 | 20.548000 |
| H | 43.995000 | 28.414000 | 19.869000 |
| H | 37.443000 | 33.221000 | 12.554000 |
| H | 37.699000 | 24.913000 | 12.451000 |
| H | 43.291000 | 23.653000 | 17.155000 |
| H | 43.958000 | 25.088000 | 17.800000 |
| H | 45.768000 | 30.774000 | 19.158000 |
| H | 46.154000 | 29.038000 | 19.129000 |
| H | 36.116000 | 34.991000 | 13.564000 |
| H | 37.010000 | 34.795000 | 15.111000 |
| H | 35.296000 | 24.716000 | 12.125000 |
| H | 35.075000 | 26.442000 | 12.547000 |
| H | 41.410000 | 23.720000 | 18.757000 |
| H | 41.989000 | 25.267000 | 19.388000 |
| H | 43.557000 | 27.246000 | 18.110000 |
| H | 41.592000 | 33.189000 | 16.972000 |
| H | 37.480000 | 30.978000 | 12.663000 |
| H | 39.262000 | 25.022000 | 14.012000 |
| H | 43.736000 | 32.214000 | 19.936000 |
| H | 42.727000 | 33.213000 | 18.906000 |
| H | 44.368000 | 32.814000 | 18.406000 |
| H | 40.783000 | 34.974000 | 15.695000 |
| H | 39.283000 | 34.846000 | 16.580000 |
| H | 39.302000 | 35.398000 | 14.917000 |
| H | 36.209000 | 27.962000 | 10.871000 |
| H | 36.526000 | 29.643000 | 11.252000 |
| H | 35.346000 | 28.745000 | 12.210000 |
| H | 40.980000 | 23.244000 | 14.531000 |
| H | 39.833000 | 23.316000 | 15.841000 |
| H | 41.458000 | 22.804000 | 16.147000 |
| **oxy-emsHb** | | | |
| N | 40.479000 | 28.259000 | 26.213000 |
| C | 41.098000 | 28.415000 | 24.886000 |
| C | 40.404000 | 29.499000 | 24.043000 |
| O | 41.061000 | 30.368000 | 23.468000 |
| C | 41.131000 | 27.044000 | 24.191000 |
| C | 42.261000 | 26.840000 | 23.208000 |
| C | 42.409000 | 25.724000 | 22.441000 |
| N | 43.382000 | 27.663000 | 23.027000 |
| C | 44.164000 | 27.015000 | 22.142000 |
| N | 43.609000 | 25.852000 | 21.784000 |
| H | 39.826000 | 27.494000 | 26.362000 |
| H | 42.129000 | 28.740000 | 25.023000 |
| H | 40.180000 | 26.866000 | 23.687000 |
| H | 41.245000 | 26.268000 | 24.950000 |
| H | 41.740000 | 24.877000 | 22.392000 |
| H | 45.122000 | 27.354000 | 21.773000 |
| H | 44.034000 | 25.157000 | 21.159000 |
| N | 45.922000 | 28.898000 | 11.058000 |
| C | 44.785000 | 28.138000 | 10.533000 |
| C | 45.050000 | 27.539000 | 9.142000 |
| O | 44.800000 | 26.351000 | 8.936000 |
| C | 43.517000 | 29.006000 | 10.568000 |
| C | 43.063000 | 29.377000 | 11.966000 |
| C | 42.118000 | 28.742000 | 12.733000 |
| N | 43.534000 | 30.433000 | 12.708000 |
| C | 42.897000 | 30.436000 | 13.888000 |
| N | 42.001000 | 29.418000 | 13.970000 |
| H | 45.791000 | 29.876000 | 11.285000 |
| H | 44.618000 | 27.291000 | 11.192000 |
| H | 42.717000 | 28.458000 | 10.074000 |
| H | 43.688000 | 29.919000 | 9.994000 |
| H | 44.244000 | 31.103000 | 12.411000 |
| H | 41.558000 | 27.865000 | 12.428000 |
| H | 43.085000 | 31.172000 | 14.663000 |
| C | 44.326000 | 29.754000 | 19.625000 |
| C | 37.670000 | 33.539000 | 13.479000 |
| C | 37.295000 | 25.739000 | 12.534000 |
| C | 43.098000 | 24.707000 | 17.773000 |
| N | 41.907000 | 29.954000 | 16.810000 |
| C | 45.825000 | 29.936000 | 19.372000 |
| C | 36.945000 | 34.560000 | 13.978000 |
| C | 35.968000 | 25.571000 | 12.371000 |
| C | 42.291000 | 24.217000 | 18.986000 |
| N | 39.924000 | 31.100000 | 15.037000 |
| C | 46.511000 | 29.955000 | 20.715000 |
| C | 43.101000 | 23.460000 | 20.011000 |
| N | 41.207000 | 27.295000 | 15.882000 |
| C | 42.865000 | 27.834000 | 17.621000 |
| C | 41.378000 | 32.356000 | 16.579000 |
| C | 38.230000 | 30.545000 | 13.341000 |
| C | 39.698000 | 26.032000 | 14.393000 |
| C | 43.595000 | 32.662000 | 18.740000 |
| C | 39.804000 | 34.793000 | 15.488000 |
| C | 36.427000 | 28.639000 | 11.685000 |
| C | 41.062000 | 23.582000 | 15.656000 |
| C | 42.746000 | 29.218000 | 17.628000 |
| C | 40.371000 | 32.269000 | 15.624000 |
| C | 38.336000 | 29.159000 | 13.346000 |
| C | 40.704000 | 26.127000 | 15.339000 |
| O | 46.759000 | 28.872000 | 21.277000 |
| O | 42.509000 | 22.658000 | 20.763000 |
| C | 43.431000 | 30.122000 | 18.464000 |
| C | 39.616000 | 33.337000 | 15.103000 |
| C | 37.533000 | 28.254000 | 12.632000 |
| C | 41.360000 | 25.042000 | 15.947000 |
| O | 46.521000 | 31.023000 | 21.357000 |
| O | 44.324000 | 23.705000 | 20.126000 |
| C | 43.078000 | 31.378000 | 18.121000 |
| C | 38.713000 | 32.834000 | 14.218000 |
| C | 37.907000 | 26.990000 | 12.953000 |
| C | 42.240000 | 25.535000 | 16.844000 |
| C | 42.096000 | 31.298000 | 17.115000 |
| C | 38.928000 | 31.435000 | 14.141000 |
| C | 39.000000 | 27.089000 | 13.840000 |
| C | 42.157000 | 26.945000 | 16.822000 |
| N | 39.234000 | 28.431000 | 14.106000 |
| Fe | 40.515000 | 29.189000 | 15.522000 |
| H | 44.029000 | 30.383000 | 20.465000 |
| H | 44.140000 | 28.728000 | 19.934000 |
| H | 37.466000 | 33.190000 | 12.500000 |
| H | 37.948000 | 24.919000 | 12.383000 |
| H | 43.515000 | 23.861000 | 17.244000 |
| H | 43.960000 | 25.273000 | 18.097000 |
| H | 46.017000 | 30.880000 | 18.868000 |
| H | 46.210000 | 29.112000 | 18.776000 |
| H | 36.185000 | 35.038000 | 13.414000 |
| H | 37.087000 | 34.920000 | 14.962000 |
| H | 35.553000 | 24.647000 | 12.072000 |
| H | 35.272000 | 26.351000 | 12.546000 |
| H | 41.493000 | 23.564000 | 18.639000 |
| H | 41.845000 | 25.074000 | 19.486000 |
| H | 43.564000 | 27.412000 | 18.296000 |
| H | 41.600000 | 33.321000 | 16.955000 |
| H | 37.509000 | 30.963000 | 12.690000 |
| H | 39.419000 | 25.055000 | 14.091000 |
| H | 44.363000 | 32.479000 | 19.491000 |
| H | 42.788000 | 33.203000 | 19.221000 |
| H | 44.029000 | 33.302000 | 17.976000 |
| H | 40.849000 | 35.050000 | 15.564000 |
| H | 39.340000 | 35.005000 | 16.440000 |
| H | 39.382000 | 35.462000 | 14.754000 |
| H | 36.336000 | 27.909000 | 10.881000 |
| H | 36.651000 | 29.599000 | 11.236000 |
| H | 35.478000 | 28.709000 | 12.211000 |
| H | 41.104000 | 23.374000 | 14.597000 |
| H | 40.075000 | 23.307000 | 15.998000 |
| H | 41.755000 | 22.909000 | 16.139000 |
| O | 39.209000 | 29.070000 | 16.878000 |
| O | 39.113000 | 28.058000 | 17.450000 |
| **deoxy-wtHb** | | | |
| N | 41.566000 | 27.516000 | 24.505000 |
| C | 41.678000 | 28.242000 | 23.233000 |
| C | 40.668000 | 29.388000 | 23.113000 |
| O | 41.015000 | 30.447000 | 22.599000 |
| C | 41.559000 | 27.273000 | 22.051000 |
| C | 42.749000 | 26.366000 | 21.856000 |
| C | 42.716000 | 25.193000 | 21.165000 |
| N | 44.072000 | 26.632000 | 22.234000 |
| C | 44.796000 | 25.603000 | 21.755000 |
| N | 44.009000 | 24.726000 | 21.116000 |
| H | 40.815000 | 26.848000 | 24.623000 |
| H | 42.660000 | 28.706000 | 23.168000 |
| H | 41.449000 | 27.857000 | 21.136000 |
| H | 40.660000 | 26.666000 | 22.171000 |
| H | 41.842000 | 24.741000 | 20.719000 |
| H | 45.873000 | 25.508000 | 21.831000 |
| H | 44.335000 | 23.869000 | 20.653000 |
| N | 45.221000 | 28.587000 | 10.618000 |
| C | 44.059000 | 28.119000 | 9.839000 |
| C | 44.464000 | 27.519000 | 8.490000 |
| O | 43.815000 | 26.568000 | 8.056000 |
| C | 43.041000 | 29.250000 | 9.654000 |
| C | 42.249000 | 29.546000 | 10.898000 |
| C | 40.967000 | 29.160000 | 11.176000 |
| N | 42.690000 | 30.316000 | 11.948000 |
| C | 41.695000 | 30.431000 | 12.839000 |
| N | 40.635000 | 29.726000 | 12.393000 |
| H | 45.450000 | 29.575000 | 10.624000 |
| H | 43.569000 | 27.312000 | 10.385000 |
| H | 42.338000 | 28.981000 | 8.868000 |
| H | 43.561000 | 30.153000 | 9.331000 |
| H | 43.611000 | 30.754000 | 11.994000 |
| H | 40.321000 | 28.570000 | 10.539000 |
| H | 41.738000 | 31.032000 | 13.741000 |
| H | 39.730000 | 29.645000 | 12.864000 |
| C | 44.579000 | 29.107000 | 19.524000 |
| C | 37.558000 | 33.090000 | 13.916000 |
| C | 37.036000 | 25.241000 | 12.959000 |
| C | 43.723000 | 24.215000 | 17.015000 |
| N | 41.948000 | 29.387000 | 16.897000 |
| C | 45.998000 | 28.811000 | 19.022000 |
| C | 36.997000 | 34.215000 | 14.397000 |
| C | 35.715000 | 24.972000 | 12.953000 |
| C | 43.238000 | 23.123000 | 17.979000 |
| N | 39.818000 | 30.566000 | 15.346000 |
| C | 46.903000 | 28.484000 | 20.209000 |
| C | 44.373000 | 22.262000 | 18.526000 |
| N | 41.273000 | 26.800000 | 15.867000 |
| C | 42.993000 | 27.254000 | 17.568000 |
| C | 41.515000 | 31.802000 | 16.635000 |
| C | 37.860000 | 30.053000 | 13.939000 |
| C | 39.710000 | 25.592000 | 14.390000 |
| C | 43.747000 | 32.047000 | 18.795000 |
| C | 40.012000 | 34.281000 | 15.589000 |
| C | 35.886000 | 28.167000 | 12.440000 |
| C | 41.504000 | 23.188000 | 15.037000 |
| C | 42.836000 | 28.632000 | 17.648000 |
| C | 40.400000 | 31.736000 | 15.809000 |
| C | 37.988000 | 28.669000 | 13.878000 |
| C | 40.829000 | 25.671000 | 15.206000 |
| O | 47.374000 | 29.422000 | 20.886000 |
| O | 44.938000 | 21.439000 | 17.765000 |
| C | 43.581000 | 29.516000 | 18.459000 |
| C | 39.655000 | 32.830000 | 15.324000 |
| C | 37.125000 | 27.769000 | 13.218000 |
| C | 41.672000 | 24.598000 | 15.554000 |
| O | 46.979000 | 27.304000 | 20.605000 |
| O | 44.650000 | 22.375000 | 19.738000 |
| C | 43.221000 | 30.780000 | 18.151000 |
| C | 38.614000 | 32.346000 | 14.593000 |
| C | 37.618000 | 26.511000 | 13.382000 |
| C | 42.599000 | 25.049000 | 16.427000 |
| C | 42.198000 | 30.722000 | 17.176000 |
| C | 38.716000 | 30.929000 | 14.585000 |
| C | 38.837000 | 26.624000 | 14.095000 |
| C | 42.338000 | 26.419000 | 16.671000 |
| N | 39.016000 | 27.945000 | 14.460000 |
| Fe | 40.462000 | 28.656000 | 15.720000 |
| H | 44.642000 | 29.908000 | 20.255000 |
| H | 44.200000 | 28.228000 | 20.042000 |
| H | 37.209000 | 32.689000 | 12.999000 |
| H | 37.716000 | 24.480000 | 12.671000 |
| H | 44.272000 | 23.750000 | 16.208000 |
| H | 44.447000 | 24.839000 | 17.519000 |
| H | 46.386000 | 29.684000 | 18.496000 |
| H | 45.975000 | 27.962000 | 18.338000 |
| H | 36.225000 | 34.724000 | 13.878000 |
| H | 37.284000 | 34.638000 | 15.325000 |
| H | 35.339000 | 24.026000 | 12.655000 |
| H | 34.986000 | 25.678000 | 13.259000 |
| H | 42.525000 | 22.469000 | 17.480000 |
| H | 42.711000 | 23.602000 | 18.803000 |
| H | 43.705000 | 26.812000 | 18.218000 |
| H | 41.838000 | 32.767000 | 16.927000 |
| H | 37.028000 | 30.474000 | 13.437000 |
| H | 39.480000 | 24.637000 | 13.995000 |
| H | 43.122000 | 32.322000 | 19.639000 |
| H | 43.751000 | 32.878000 | 18.096000 |
| H | 44.773000 | 31.915000 | 19.140000 |
| H | 41.076000 | 34.449000 | 15.507000 |
| H | 39.706000 | 34.582000 | 16.581000 |
| H | 39.545000 | 34.951000 | 14.884000 |
| H | 35.646000 | 27.416000 | 11.689000 |
| H | 36.037000 | 29.109000 | 11.916000 |
| H | 35.037000 | 28.265000 | 13.114000 |
| H | 42.363000 | 22.563000 | 15.217000 |
| H | 41.315000 | 23.168000 | 13.976000 |
| H | 40.664000 | 22.715000 | 15.526000 |
| **oxy-wtHb** | | | |
| N | 41.398000 | 27.584000 | 24.735000 |
| C | 41.518000 | 28.258000 | 23.435000 |
| C | 40.533000 | 29.418000 | 23.266000 |
| O | 40.913000 | 30.467000 | 22.751000 |
| C | 41.375000 | 27.235000 | 22.309000 |
| C | 42.595000 | 26.367000 | 22.150000 |
| C | 42.608000 | 25.006000 | 22.195000 |
| N | 43.852000 | 26.827000 | 21.749000 |
| C | 44.574000 | 25.720000 | 21.493000 |
| N | 43.866000 | 24.620000 | 21.792000 |
| H | 40.624000 | 26.949000 | 24.880000 |
| H | 42.511000 | 28.695000 | 23.343000 |
| H | 41.232000 | 27.766000 | 21.367000 |
| H | 40.495000 | 26.612000 | 22.481000 |
| H | 41.764000 | 24.361000 | 22.393000 |
| H | 45.550000 | 25.704000 | 21.016000 |
| H | 44.133000 | 23.680000 | 21.468000 |
| N | 45.226000 | 28.395000 | 10.703000 |
| C | 44.044000 | 28.086000 | 9.895000 |
| C | 44.417000 | 27.559000 | 8.510000 |
| O | 43.776000 | 26.614000 | 8.052000 |
| C | 43.159000 | 29.332000 | 9.773000 |
| C | 42.411000 | 29.659000 | 11.036000 |
| C | 41.123000 | 29.307000 | 11.311000 |
| N | 42.903000 | 30.418000 | 12.094000 |
| C | 41.902000 | 30.527000 | 12.972000 |
| N | 40.817000 | 29.874000 | 12.524000 |
| H | 45.354000 | 29.355000 | 11.014000 |
| H | 43.471000 | 27.301000 | 10.388000 |
| H | 42.426000 | 29.174000 | 8.983000 |
| H | 43.772000 | 30.188000 | 9.489000 |
| H | 40.463000 | 28.741000 | 10.673000 |
| H | 41.960000 | 31.091000 | 13.894000 |
| H | 39.907000 | 29.872000 | 12.976000 |
| C | 44.385000 | 29.666000 | 19.385000 |
| C | 37.144000 | 32.794000 | 13.472000 |
| C | 37.153000 | 24.898000 | 13.072000 |
| C | 43.911000 | 24.626000 | 17.194000 |
| N | 41.742000 | 29.635000 | 16.756000 |
| C | 45.808000 | 29.435000 | 18.864000 |
| C | 36.403000 | 33.847000 | 13.870000 |
| C | 35.851000 | 24.551000 | 13.038000 |
| C | 43.445000 | 23.761000 | 18.379000 |
| N | 39.525000 | 30.549000 | 15.152000 |
| C | 46.701000 | 28.957000 | 20.008000 |
| C | 44.563000 | 23.185000 | 19.253000 |
| N | 41.307000 | 26.939000 | 15.827000 |
| C | 43.015000 | 27.637000 | 17.459000 |
| C | 41.014000 | 31.979000 | 16.496000 |
| C | 37.687000 | 29.805000 | 13.693000 |
| C | 39.795000 | 25.509000 | 14.503000 |
| C | 43.227000 | 32.477000 | 18.678000 |
| C | 39.307000 | 34.267000 | 15.302000 |
| C | 35.826000 | 27.712000 | 12.374000 |
| C | 41.790000 | 23.286000 | 15.303000 |
| C | 42.712000 | 28.992000 | 17.511000 |
| C | 39.953000 | 31.782000 | 15.620000 |
| C | 37.895000 | 28.432000 | 13.755000 |
| C | 40.929000 | 25.729000 | 15.272000 |
| O | 47.224000 | 29.794000 | 20.773000 |
| O | 45.724000 | 23.617000 | 19.115000 |
| C | 43.346000 | 29.960000 | 18.319000 |
| C | 39.130000 | 32.781000 | 15.059000 |
| C | 37.084000 | 27.436000 | 13.176000 |
| C | 41.856000 | 24.748000 | 15.682000 |
| O | 46.712000 | 27.744000 | 20.294000 |
| O | 44.213000 | 22.411000 | 20.172000 |
| C | 42.832000 | 31.171000 | 18.018000 |
| C | 38.201000 | 32.179000 | 14.269000 |
| C | 37.648000 | 26.223000 | 13.431000 |
| C | 42.757000 | 25.333000 | 16.502000 |
| C | 41.821000 | 30.991000 | 17.044000 |
| C | 38.435000 | 30.781000 | 14.328000 |
| C | 38.857000 | 26.461000 | 14.136000 |
| C | 42.407000 | 26.697000 | 16.636000 |
| N | 38.960000 | 27.817000 | 14.390000 |
| Fe | 40.393000 | 28.730000 | 15.527000 |
| H | 44.412000 | 30.486000 | 20.100000 |
| H | 44.063000 | 28.778000 | 19.927000 |
| H | 36.946000 | 32.346000 | 12.532000 |
| H | 37.888000 | 24.168000 | 12.848000 |
| H | 44.447000 | 24.005000 | 16.490000 |
| H | 44.645000 | 25.340000 | 17.534000 |
| H | 46.201000 | 30.360000 | 18.440000 |
| H | 45.789000 | 28.670000 | 18.090000 |
| H | 35.638000 | 34.258000 | 13.262000 |
| H | 36.535000 | 34.302000 | 14.816000 |
| H | 35.541000 | 23.570000 | 12.778000 |
| H | 35.072000 | 25.226000 | 13.283000 |
| H | 42.841000 | 22.937000 | 17.999000 |
| H | 42.809000 | 24.377000 | 19.015000 |
| H | 43.791000 | 27.293000 | 18.094000 |
| H | 41.207000 | 32.976000 | 16.793000 |
| H | 36.863000 | 30.135000 | 13.118000 |
| H | 39.619000 | 24.512000 | 14.195000 |
| H | 42.707000 | 32.588000 | 19.624000 |
| H | 42.997000 | 33.335000 | 18.052000 |
| H | 44.299000 | 32.496000 | 18.873000 |
| H | 40.351000 | 34.545000 | 15.301000 |
| H | 38.886000 | 34.552000 | 16.254000 |
| H | 38.831000 | 34.860000 | 14.535000 |
| H | 35.652000 | 26.910000 | 11.663000 |
| H | 35.921000 | 28.629000 | 11.797000 |
| H | 34.965000 | 27.791000 | 13.037000 |
| H | 42.713000 | 22.761000 | 15.500000 |
| H | 41.564000 | 23.147000 | 14.259000 |
| H | 41.022000 | 22.785000 | 15.875000 |
| O | 38.863000 | 28.259000 | 17.696000 |
| O | 39.554000 | 27.781000 | 18.562000 |
